# Predictors and outcomes of mycobacteremia among HIV-infected smear- negative presumptive tuberculosis patients in Uganda

**DOI:** 10.1186/s12879-015-0812-4

**Published:** 2015-02-15

**Authors:** Lydia Nakiyingi, Willy Ssengooba, Damalie Nakanjako, Derek Armstrong, Molly Holshouser, Bruce J Kirenga, Maunank Shah, Harriet Mayanja-Kizza, Moses L Joloba, Jerrold J Ellner, Susan E Dorman, Yukari C Manabe

**Affiliations:** Infectious Diseases Institute, Makerere University College of Health Sciences, Kampala, Uganda; Makerere University College of Heath Sciences, Kampala, Uganda; Academic Medical Centre, University of Amsterdam, Amsterdam, The Netherlands; Johns Hopkins University School of Medicine, Baltimore, MD USA; Boston Medical Center, Boston University School of Medicine, Boston, MA USA

**Keywords:** Predictors, Mortality, Mycobacterial infections, Bacteremia, Smear- negative, HIV, LAM, Sub-Saharan Africa

## Abstract

**Background:**

Sputum smear microscopy for tuberculosis (TB) diagnosis lacks sensitivity in HIV-infected symptomatic patients and increases the likelihood that mycobacterial infections particularly disseminated TB will be missed; delays in diagnosis can be fatal. Given the duration for MTB growth in blood culture, clinical predictors of MTB bacteremia may improve early diagnosis of mycobacteremia. We describe the predictors and mortality outcome of mycobacteremia among HIV-infected sputum smear-negative presumptive TB patients in a high prevalence HIV/TB setting.

**Methods:**

Between January and November 2011, all consenting HIV-infected adults suspected to have TB (presumptive TB) were consecutively enrolled. Diagnostic assessment included sputum smear microscopy, urine Determine TB lipoarabinomannan (LAM) antigen test, mycobacterial sputum and blood cultures, chest X-ray, and CD4 cell counts in addition to clinical and socio-demographic data. Patients were followed for 12 months post-enrolment.

**Results:**

Of 394 sputum smear-negative participants [female, 63.7%; median age (IQR) 32 (28–39) years], 41/394 (10.4%) had positive mycobacterial blood cultures (mycobacteremia); all isolates were *M. tuberculosis* (MTB). The median CD4 cell count was significantly lower among patients with mycobacteremia when compared with those without (CD4 31 versus 122 cells/μL, p < 0.001). In a multivariate analysis, male gender [OR 3.4, 95%CI (1.4-7.6), p = 0.005], CD4 count <100 cells/μL [OR 3.1, 95% CI (1.1-8.6), p = 0.030] and a positive lateral flow urine TB LAM antigen test [OR 15.3, 95%CI (5.7-41.1), p < 0.001] were significantly associated with mycobacteremia. At 12 months of follow-up, a trend towards increased mortality was observed in patients that were MTB blood culture positive (35.3%) compared with those that were MTB blood culture negative (23.3%) (p = 0.065).

**Conclusions:**

Mycobacteremia occurred in 10% of smear-negative patients and was associated with higher mortality compared with smear-negative patients without mycobacteremia. Advanced HIV disease (CD4 < 100 cells/mm^3^), male gender and positive lateral flow urine TB LAM test predicted mycobacteremia in HIV-infected smear-negative presumptive TB patients in this high prevalence TB/HIV setting.

## Background

Tuberculosis (TB) incidence and mortality have increased dramatically because of the human immunodeficiency virus (HIV) epidemic. TB is the leading cause of death among HIV-infected persons in sub-Saharan Africa (SSA) [1-4]. Sputum smear microscopy for acid-fast bacilli (AFB), the most widely used TB diagnostic method, detects less than half of HIV-infected TB cases [5,6]. Sputum smear-negative pulmonary TB [7,8] is associated with delayed diagnosis and treatment with subsequently high mortality [9-11] especially in TB-HIV co-infected patients. Mortality in sputum smear-negative HIV-infected patients is likely due in part to undiagnosed mycobacterial disease including mycobacteremia, an often fatal form of disseminated TB.

The prevalence of mycobacteremia among HIV-infected hospitalized febrile patients ranges from 9 to 22% [12-15]. In Uganda, mycobacteremia has been reported in 23% of hospitalized HIV-infected patients with sepsis, half of whom die within a month of admission [16]. Mortality in patients with mycobacteremia often results from delayed or missed TB diagnosis. Early diagnosis and treatment therefore has the potential to reduce mycobacteremia attributable mortality [17]. However, diagnosis of mycobacteremia is often delayed due to atypical presentation, which makes clinical diagnosis more difficult [18-21]. The high cost of TB blood culture and the need for sophisticated laboratory infrastructure make the test unaffordable for routine patient care in high prevalence HIV/TB resource-limited settings [22]. In addition, TB blood culture has a minimum turnaround time of 3 weeks [23,24] which further contributes to delayed diagnosis and subsequent morbidity and mortality. Death occurs before TB blood culture results in the majority of patients [14]. Given the duration required for MTB growth in blood culture, clinical predictors of MTB bacteremia and rapid diagnostic tests may improve early diagnosis of mycobacteremia particularly in settings with increased HIV /TB co infection. Mortality in HIV- infected, smear-negative TB patients could be reduced through early identification and treatment of patients who have MTB bacteremia.

We have previously shown that positive TB blood culture was associated with positive urine TB lipoarabinomannan (LAM) antigen test [25]. As a rapid point-of-care test, the urine TB LAM test, when used with other clinical factors, could contribute to early MTB bacteremia diagnosis and rapid initiation of anti-tuberculosis therapy. We report the predictors and outcomes of mycobacteremia among HIV-infected sputum smear-negative presumptive TB patients in a high prevalence HIV/TB setting.

### Ethical considerations

This study was nested within a TB diagnostic study that was approved by the Scientific Review Board of the Infectious Diseases Institute (IDI), the Institutional Review Boards of the Joint Clinical Research Centre, Kampala, Uganda, Johns Hopkins University, Baltimore, USA, and the Uganda National Council for Science and Technology. Participants provided informed consent.

## Methods

### Study design and setting

The analysis was performed on data collected from participants that were prospectively enrolled in a TB diagnostic study that assessed the accuracy of the lateral flow urine Determine TB LAM Ag test (henceforth called the ‘TB LAM’ test) for the diagnosis of TB among HIV-infected TB suspects [25]. Participants were recruited from the outpatient adult Infectious Diseases Institute (IDI) Clinic [26] and the inpatient wards of the Mulago National Referral Hospital, Kampala, Uganda between January 2011 and November 2011.

### Patients recruitment

HIV-infected patients clinically suspected to have TB (presumptive TB patients) aged ≥18 years were recruited. A patient was clinically suspected to have TB if he or she reported having cough, fever, night sweats, or weight loss. Patients were excluded if they had taken anti-TB medication for more than two days within 60 days prior to enrolment. At enrolment, participants were interviewed to obtain socio-demographic and medical information before study specific specimens were collected. The participants provided two spot sputum samples for direct fluorescence microscopy (FM) and Ziehl-Neelsen (ZN) microscopy; mycobacterial growth indicator tube (MGIT) and Lowenstein-Jensen (LJ) sputum cultures. Sputum induction using 7% nebulized hypertonic saline was performed for participants who were unable to spontaneously expectorate sputum. Blood for mycobacterial blood cultures and CD4 cell count was collected from each participant using aseptic techniques. Species identification on all positive TB cultures was performed. Urine was collected for TB LAM testing. Chest radiographs were obtained for all male participants and non-pregnant women. Further details on patient recruitment were described elsewhere [25]. The analysis included participants who were AFB smear-negative on both ZN and FM microscopy and had TB blood culture results available.

### Laboratory procedures

After collection, sputum specimens were kept in a cool box until they were transferred to a refrigerator upon receipt in the mycobacteriology (BSL-3) laboratory under Makerere University, Department of Medical Microbiology located within the Mulago Hospital complex. Smears were prepared from each unprocessed sputum specimen and stained using ZN and auramine-O methods. The remaining portions of the sputum specimens were decontaminated using N-acetyl-L-cysteine-NaOH of which 0.5 ml was used to inoculate MGIT culture using the MGIT 960 system (Becton and Dickinson, Franklin Lakes, NJ USA) and 0.1 ml to inoculate LJ culture media. Cultures were incubated at 37°C for up to six weeks for MGIT and eight weeks for the LJ method.

MYCO/F LYTIC (Becton and Dickinson) tubes were used for mycobacterial blood cultures; blood inoculum volume was 3 ml and incubated in Bactec 9120 machine (Becton and Dickinson, Franklin Lakes, NJ USA) for up to six weeks. Positive cultures were assessed for presence of AFB using ZN staining and light microscopy, and for *M. tuberculosis* (MTB) complex using an anti MPB64 antibody assay (Capilia TB-Neo, TAUNS Laboratories, Numazu, Japan). Mycobacteremia was defined as isolation of mycobacteria from the mycobacterial blood culture.

For the TB LAM test, 60 μl was pipetted onto the sample pad. According to the manufacturer’s instructions, the strip was read 25 minutes later by two different technicians independently who compared the test strips with the reference card provided by the manufacturer and graded the result from 1+ to 5+. A result was considered positive if the band was graded as 2+ or above.

CD4 cell count was performed at a certified laboratory at the IDI [26] following the laboratory standard procedure.

All study TB laboratory results (except for the urine TB LAM test that was an investigational test) were made available to the attending clinicians. Discharged participants were contacted by telephone to deliver TB results and those whose TB tests were positive were requested to return for TB treatment. During the phone interviews, participants were also asked if TB treatment had been initiated. Participants whose TB results were positive but could not be contacted by telephone had study home visits performed during which, information on TB treatment and survival status was obtained. MTB-positive patients (sputum smear positive by any of the methods or sputum culture positive by any method or blood culture positive) were immediately initiated on TB treatment by the attending clinician according to the guidelines from the Uganda Ministry of Health TB and Leprosy program [27].

### Assessment of mortality

Information on survival status was obtained during monthly phone interviews that were conducted for a period up to 12 months post- enrolment. For patients who died in the hospital, the date of death was recorded. For patients who were discharged, they or their family were contacted by mobile phone at least monthly after enrolment to obtain survival status. For those who died, the date of death was recorded; if the exact date of death was not available, the date of death was recorded as the date of the follow-up phone call.

### Data management and statistical analysis

Smear-negative participants were primarily stratified according to their TB blood culture status reported as either positive or negative. Continuous variables were summarized using medians and inter-quartile ranges (IQR) while categorical variables were summarized using frequencies, proportions and percentages. Using Wilcoxon rank sum test for continuous variables and Chi-square test or Fisher’s exact test for categorical variables, we compared the characteristics of the study population stratified by TB blood culture status.

To identify predictors of mycobacteremia among sputum smear-negative HIV-infected presumptive TB patients, a multivariate logistic regression model was constructed using all factors from bivariate analysis that had a p-value of ≤0.2 and those of known clinical significance, specifically chest X-ray (CXR) findings. Using a systematic backward elimination approach, non-significant variables were removed from the model until no further variables were eligible for removal to arrive at the final parsimonious model. A p-value of <0.05 in the final model was considered statistically significant.

In the assessment of mortality, the endpoint was mortality 12 months post- enrolment. Kaplan–Meier estimates of mortality between participants with and without mycobacteremia were compared using the log-rank test.

All data were analyzed using STATA® version 12.0 (StataCorp, 4905 Lakeway Drive College Station, Texas USA).

## Results

### Study participants’ characteristics

**Figure 1:**
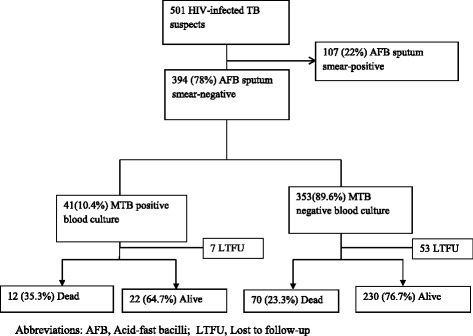
**Participant flow diagram.**

Of the 501 TB suspects who provided blood for TB blood culture testing, 394 were sputum smear-negative and eligible for the analysis (Figure 1); 63.7% were female with a median age (IQR) and CD4 cell count (IQR) of 32 (28–39) years and 106 (24–308) cells/mm^3^, respectively (Table 1). Of the 68.5% (270/394) who were hospitalized, the median CD4 was lower compared to the non-hospitalized participants [59 cells/μL (IQR 14–182) versus 281 cells/μL (IQR 106–491), respectively, p < 0.001].

**Table 1:** **Comparing characteristics of sputum smear-negative HIV-infected presumptive TB patients with and without mycobacteremia, N = 394**

**Characteristics**	**Total, N = 394**	**Positive TB blood culture N = 41**	**Negative TB blood culture N = 353**	**P value**
Median age(IQR) years	32(28–39)	35(28–40)	32(27–39)	0.260
Male, # (%)	**143(36.3)**	**24(58.5)**	**119(33.7)**	**0.002**
Hospitalization	**270(68.5)**	**36(87.8)**	**234(66.3)**	**0.005**
Median CD4 cells/mm^3^ (IQR)	**106(24–308)**	**31(10–73)**	**122(28–323)**	**<0.001**
CD4 < 100	**190(48.2)**	**35(85.4)**	**155(43.9)**	**<0.001**
Not on ART, # (%)	246(62.4)	31(75.6)	215(60.9)	0.070
**CXR abnormal, # (%),N = 361*	209(57.9%)	21(61.8%)	188(57.5%)	0.631
Sputum culture positive (MGIT/or LJ)	**88(22.3)**	**31(75.6)**	**57(16.1)**	**<0.001**
*Positive urine TB LAM test (N = 393)	**52(13.2)**	**29(70.7)**	**23(6.5)**	**<0.001**

*Means the number of variables is less than the total N = 394 because of missing data due to unperformed study tests.

**CXR was read by physician and reported as ‘abnormal and suggestive of TB’.

Abbreviations: *CXR* chest X-ray, *LAM* Lipoarabinomannan, *ART* Antiretroviral therapy, *IQR* Inter-quartile range.

### Mycobacteremia in study population

Of the 394 sputum smear-negative participants, 41/394 (10.4%) had positive mycobacterial blood cultures, and all cultured isolates were *M. tuberculosis* (MTB) complex on species identification. The average time to positivity for the MTB positive blood cultures was 24(±8SD) days. All 41 participants with positive MTB blood cultures reported excessive weight loss (i.e. weight loss of >10% in four weeks) and the majority, 31/41(75.6%) had one or more sputum cultures positive for MTB on LJ and/ or MGIT culture. Among the 10 MTB blood culture-positive, sputum culture-negative participants, all (10/10, 100%) were hospitalized, had a normal CXR and 5/10 (50%) had CD4 cell count < 50cells/mm^3^ [median CD4 (IQR) =50 (7–137) cells/mm^3^]. Urine TB LAM test was positive in 70.7% (29/ 41) of the MTB blood culture-positive participants.

### Predictors of mycobacteremia in HIV-infected smear-negative participants

Results of the bivariate analysis are summarized in Table 2. On multivariate logistic regression analysis, male gender [OR 3.4, 95%CI (1.4-7.9), p = 0.005], CD4 cell count <100 cells/μL [OR 3.1, 95%CI (1.1-8.6), p = 0.030] and a positive urine TB LAM test [OR 15.3, 95% CI (5.7-41.1), p < 0.001] were independently associated with mycobacteremia in HIV-infected sputum smear-negative clinically suspected TB patients.

**Table 2:** Multivariate analysis for predictors of mycobacteremia in sputum smear-negative HIV-infected presumptive TB patients*

	**Unadjusted OR**		**Adjusted OR**	
**Characteristics**		**P value**		**P value**
	**(95%CI)**		**(95%CI)**	
Age (years)	1.1(0.9-1.3)	0.260	-	-
Hospitalization	3.7(1.4-9.6)	0.005	-	-
Male gender	**2.8(1.4-5.4)**	**0.002**	**3.4(1.4-7.9)**	**0.005**
Not on ART	2.0(1.0-4.2)	0.07	1.5(0.5-4.0)	0.128
CD4 < 100	**7.5(3.1-18.2)**	**<0.001**	**3.1(1.1-8.6)**	**0.030**
**CXR abnormal	1.2(0.6-2.5)	0.631	0.8(0.3-2.0)	0.637
Positive urine TB LAM test	**34.6(15.6-76.5)**	**<0.001**	**15.3(5.7-41.1)**	**<0.001**

*N = 361 with complete records. The model adjusted for gender, CD4 cell count, CXR findings, ART therapy, and positive lateral flow urine TB LAM test.

**CXR read by physician and reported as ‘abnormal and suggestive of TB’.

Abbreviations: *CXR* chest X-ray, *LAM* Lipoarabinomannan, *ART* Antiretroviral therapy, *OR* Odds ratio, *CI* Confidence intervals.

### Mortality in participants with mycobacteremia

Follow-up information was available for 34/41 (82.9%) of the sputum smear-negative participants with positive MTB blood culture (mycobacteremia) and 300/353 (85%) of the blood culture-negative participants. The remaining participants (7 with mycobacteremia and 53 without mycobacteremia) could not be reached by phone or home visits.

**Figure 2:**
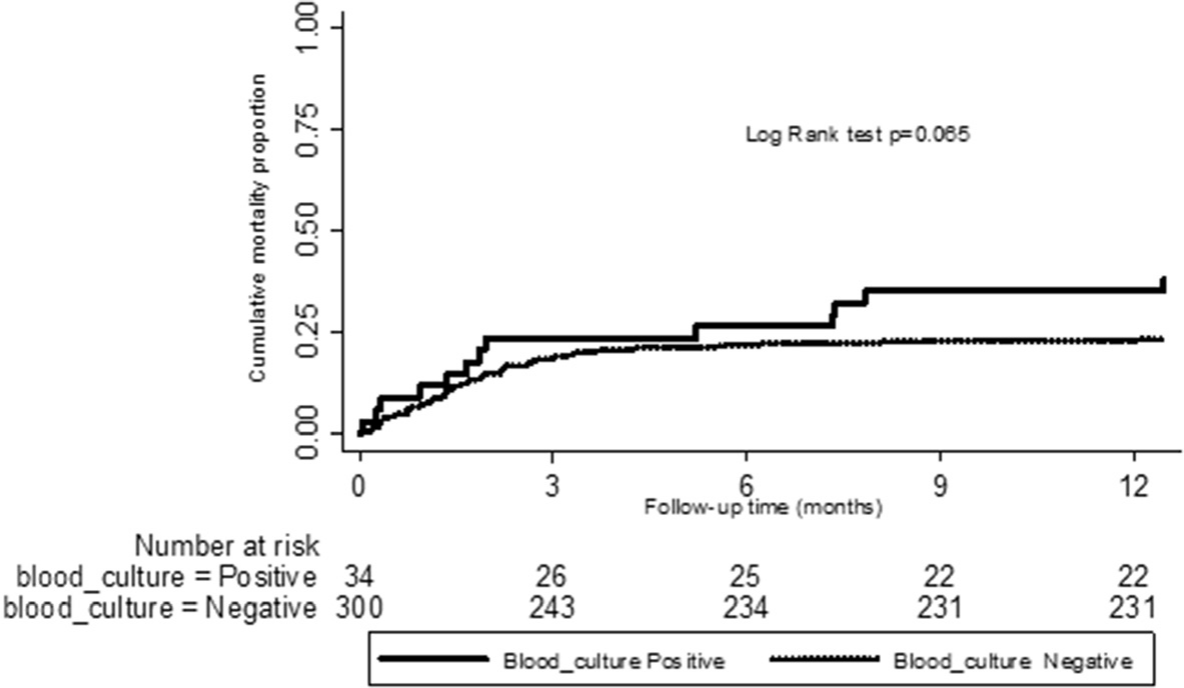
**Kaplan-Meier curve comparing mortality between participants with and without MTB bacteremia.**

At 12 months, a trend towards increased mortality existed in those that were MTB blood culture positive (35.3%, 12/34) compared to those that were MTB blood culture negative (23.3%, 70/300) although this difference did not reach statistical significance (Log rank test, p = 0.065) (Figure 2). Nearly 66.7% of all the deaths that occurred among patients with mycobacteremia occurred within the first three months of enrolment. Mortality outcome among the study participants when further stratified by urine TB LAM test results is shown in Table 3.

**Table 3:** **Summary of 12-month follow-up outcomes for HIV-infected smear-negative participants stratified by TB blood culture status**

	**MTB blood culture positive (N = 41), proportion (%)**	**Blood culture negative (N = 353), proportion (%)**
Lost to follow-up	7/41 (17)	53/353 (15)
12 months follow-up data available	34/41 (83%)	300/353 (85)
*Overall mortality	12/34 (35.3)	70/300 (23.3)
Mortality by urine LAM status**		
*Urine TB LAM positive and died	8/23 (34.8)	7/19 (36.8)
*Urine TB LAM negative and died	4/11 (36.4)	62/280 (22.1)
*TB treatment initiated	22/34 (64.7)	58/300 (19.3)
*No TB treatment	12/34 (35.3)	242/300 (80.7)
*TB treatment initiated and died	5/22 (22.7)	12/58(20.7)
*Not initiated on TB treatment and died	7/12 (58.3)	58/242(24.0)

*Proportions were calculated among participants that had follow-up data available.

**One patient did not have a LAM test performed.

Of the 34 patients with mycobacteremia that had follow-up information available, 64.7% (22/34) had been initiated on TB treatment at the time the MTB blood culture results were positive (Table 3). Compared to patients with MTB bacteremia who did not receive TB treatment, the proportion of patients who died by 12 months was significantly lower in the mycobacteremia patients who received TB treatment [22.7%, 5/22 vs. 58.3% (7/12) p = 0.0473].

## Discussion

In this high prevalence TB/HIV setting, nearly one in every ten HIV-infected smear-negative presumptive TB patients are likely to have MTB bacteremia. Advanced HIV (CD4 cell count <100cells/mm^3^), male gender and positive urine TB LAM test predicted presence of MTB bacteremia among smear-negative clinically suspected TB patients. Similar to prior reports from SSA [12,18-20,28], we found a high prevalence of mycobacteremia among TB/HIV co-infected patients whose TB was not diagnosed by routine smear microscopy. Although sputum TB culture could identify 75% of the patients with mycobacteremia in our study, this test is not routinely performed in most high TB/HIV prevalence resource-limited settings [22]. CXR, another commonly available TB diagnostic method, was reported as abnormal and suggestive of TB in only 62% of the patients with mycobacteremia. Our findings confirm earlier reports that a significant proportion of mycobacteremic patients cannot be diagnosed using routinely available radiological and laboratory tests [12,18-20,28].

We found a trend towards increased mortality in HIV-infected smear-negative patients who had mycobacteremia when compared to those without mycobacteremia even with this small sample size. Nearly two-thirds of the blood culture positive patients died within the first three months of enrolment. Blood culture services are still limited in SSA and it is unlikely that these services will become widely available in many TB/HIV prevalent resource-limited settings. We found that immunologically advanced HIV (CD4 cell count <100cells/mm^3^) and male gender were independently associated with mycobacteremia among HIV-infected sputum smear-negative patients, consistent with previous reports [15,29]. The association between male gender and mycobacteremia may be explained in part by the poorer health seeking behavior in men compared to women in SSA [30]. Many more men present with advanced immunosuppression and opportunistic infections compared to women.

Urine TB LAM was an independent predictor for mycobacteremia among HIV-infected smear negative-clinically suspected TB patients. The lateral-flow urine TB LAM test yields rapid results in 25 minutes and is very easy to perform. Therefore, urine TB LAM test could be used to predict mycobacteremia in smear-negative HIV-infected presumptive TB patients within a few hours after presentation. Early identification and treatment of smear-negative patients who are likely to have mycobacteremia could reduce attributable mortality. Further, similar to an earlier South African report [31], over two thirds of the participants with mycobacteremia had a positive urine TB LAM test. Our findings provide additional evidence that urine TB LAM test may be beneficial in patients with disseminated TB. A correlation between LAM positivity and patient outcomes has been previously published [32].

Our study had limitations. First, we did not perform autopsy studies for participants who died during the study and we were unable to establish definitively the actual cause of death in the study population. Secondly, we may not have found statistically significant differences in some of the comparisons made between the mycobacteremia and non-mycobacteremia patients due to the small sample size, particularly in the mycobacteremia group. Thirdly, mycobacteremia was assessed by inoculation of 3 ml of blood into MYCO/F LYTIC cultures on a single occasion and thus mycobacteremia in the population could have been underestimated. The yield of MTB blood cultures may have been increased substantially by testing larger volumes of blood and by sampling on more than one occasion. Lastly, we could not determine whether blood culture results had additional benefits for clinical decision-making regarding the management of the sputum smear-negative participants and we could not ascertain the basis for the clinical decisions made by the attending physicians.

Despite these limitations, we report findings from both inpatient and outpatient populations that were recruited and underwent rigorous, protocol-specified laboratory testing. Our study is among only a few studies in the field of MTB bacteremia and highlights how clinical correlates and urine TB LAM test can be utilized in clinical management of smear-negative HIV-infected patients with mycobacteremia in a resource- limited setting. We believe that our findings are reliable and generalizable to most high TB/HIV prevalence resource-limited settings.

## Conclusion

Mycobacteremia occurred in 10% of smear-negative patients and was associated with higher mortality compared with smear-negative patients without mycobacteremia. Advanced HIV disease (CD4 < 100 cells/mm^3^), male gender and positive lateral flow urine TB LAM test predicted mycobacteremia in HIV-infected smear-negative presumptive TB patients in this high prevalence TB/HIV setting.

### Funding

LN is a PhD candidate supported by by Medical Education for Equitable Services to All Ugandans a Medical Education Partnership Initiative grant number 5R24TW008886 from the Office of Global AIDS Coordinator and the U. S. Department of Health and Human Services, Health Resources and Services Administration and National Institutes of Health. The study project was supported by the Division of Microbiology and Infectious Diseases, National Institute of Allergy and Infectious Diseases, National Institutes of Health, Department of Health and Human Services (contract number HHSN2722000900050C to “TB Clinical Diagnostics Research Consortium”). Additional support was to YCM by the Johns Hopkins University Center for AIDS Research (Grant Number 1P30AI094189 from the National Institute of Allergy and Infectious Diseases). Its contents are solely the responsibility of the authors and do not necessarily represent the official views of the government.
